# 胸腔镜肺切除手术，单孔是否较多孔更微创？

**DOI:** 10.3779/j.issn.1009-3419.2018.04.13

**Published:** 2018-04-20

**Authors:** 建军 秦

**Affiliations:** 郑州大学附属肿瘤医院暨河南省肿瘤医院胸外科 Department of Thoracic Surgery, Cancer Hospital Affiliated to Zhengzhou University & Henan Tumor Hospital

随着科技的发展，“微创”这一概念已深入到外科手术的各种领域，其主要优势就是在减少手术损伤的同时实现更好的治疗效果。而手术创伤主要有三个来源，一是切口创伤，二是脏器损伤，三是全身系统性影响^[1]^。

黄麟等选择接受单孔VATS肺切除手术的非小细胞肺癌（non-small cell lung cancer, NSCLC）患者，回顾性对比分析高龄（≥65岁）与非高龄（< 65岁）患者的围术期效果，结果发现高龄组并不增加手术并发症。

单孔胸腔镜（video-assisted thoracoscopic surgery, VATS）肺切除术在国内外应用日益广泛。从2004年Rocco等^[2]^首次报道单孔VATS肺楔形切除手术开始，关于单孔与多孔手术的争论就没有停止过。一般认为，孔的减少将会减少手术创伤，但由于单孔会造成器械互相之间干扰、对术者和助手操作习惯的改变等，单孔与多孔“谁更微创”也不断引发争论。

## 1 肿瘤学效果

美国国立综合癌症网络（National Comprehensive Cancer Network, NCCN）针对NSCLC的指南推荐：只要符合外科学和肿瘤学原则，VATS或微创手术应该重点考虑用于没有解剖学禁忌和手术禁忌症的患者。在有丰富经验的高诊治量医疗中心，在特定病人中行VATS较开放手术（Open）可以提高近期疗效（如：减少疼痛、降低住院日、功能恢复更快、降低术后并发症等）^[3]^。需要指出的是，该推荐是基于多孔VATS在NSCLC治疗中的研究和长期实践经验得来的。

黄麟等的研究探讨了高龄与非高龄患者的单孔肺切除，两组清扫的淋巴结组数和个数无统计学差异。台湾Wang等^[4]^2015年发表的一篇倾向评分匹配研究发现，单孔较多孔VATS肺切除手术，清扫淋巴结数目更多。但Bao等^[5]^针对该研究发表的“letter to editor”讨论认为，这种优势可能与选择偏倚有关，且由于器械之间的干扰，单孔组可能会有更多的淋巴结破碎从而导致个数增多。Ji等^[6]^进行的多中心回顾性研究，收集了亚洲四个中心（上海交通大学附属胸科医院120例、国立台湾大学附属医院155例、香港大学圣玛丽医院及深圳医院106例、韩国大学九老医院77例）接受胸腔镜肺叶或者肺段切除的共458例患者的临床资料，根据手术方式分为单孔VATS组（*n*=166）和多孔VATS组（*n*=292）。对比单孔与多孔后发现：两组患者淋巴结清扫数目、组数没有统计学差异，证实了单孔VATS用于肺癌的可行性。

已有的证据仅仅是“VATS可能好于Open”，但对于“VATS是否优于Open”仍无确定答案。VATS方法很多，“单孔VATS优于多孔VTAS肺癌手术吗？”这个问题对于回答“VATS是否优于Open”并无帮助。胸外科医师应该更多地关注专业诊治和外科质量以改进肺癌患者的短期和长期疗效，而不应只一味争论切口^[7]^。

## 2 安全性和疼痛

黄麟等的这篇研究发现单孔用于高龄患者未增加术后总体并发症，但高龄组术后房颤和下肢深静脉血栓发病率增加。多数关于单孔对比多孔的研究，显示单孔围术期效果不劣于多孔。Wang^[4]^的研究显示单孔组手术时间更短，术中失血量更少。Ji等^[6]^的研究，对比单孔与多孔VATS肺切除手术后，结果发现：单孔组的手术时间、术中发生500 mL以上的出血次数要明显高于多孔组；术中改变手术方式在多孔组更常见，说明外科医生倾向于选择单孔径路进行相对简单的手术。

减轻疼痛是微创较开放手术最大的一个优势，Ji^[6]^的研究中，单孔组的疼痛评分在术后第一天和第二天要显著低于多孔组，但术后第三天两组疼痛无显著差异^[6]^。Young等^[8]^进行了一项专门针对单孔术后疼痛的研究，共纳入证据级别较高的10篇文章和2篇摘要，入组患者包括解剖性肺切除、肺活检和自发性气胸手术。被纳入的这些研究普遍样本量很小且都是非随机分组，结论也并不完全一致。作者总结后认为单孔较多孔可能在减轻术后72 h内的疼痛方面有微弱优势，接着的疼痛比较则没有差异。10分法视觉模拟评分（Visual Analogue Scale, VAS）显示疼痛程度改善了1分-2分，三分之一以上的研究没有发现任何有统计学意义的不同。其中两项研究比较了单孔和多孔肺叶切除平均疼痛评分和吗啡用量，结果也是没有统计学差异。因而，作者认为单孔只是没有比多孔引起更多的疼痛，至于单孔是否能够比多孔减轻疼痛则需要进一步研究。

## 3 几何学差异

由于VATS的设计理念与直观视觉效果关系密切，故VATS的几何结构几乎等同于直视下的场景。几何学是数学领域中与视觉相关的科学。传统三孔VATS的菱形几何构型使得手术器械可从不同方向聚合到病变位置，并与光源相互影响。单孔VATS从矢状面接近病变，器械与图像平面平行，使得外科医生能将手术支点带入胸腔内处理肺部病变的方法与开胸手术相似。从几何学原理看，单孔VATS似乎更易使外科医生从开放转向腔镜^[9]^。但单孔造成的器械之间互相干扰也是胸外科医师面临的困难。

## 4 机器人辅助腔镜外科

胸外科微创领域的另一项重要进展是“达芬奇机器人辅助胸腔镜外科（robotic-assisted thoracic surgery, RATS）”，经典的RATS是四孔法，与单孔相比到底哪个更微创，也是一个有意思的话题。随着设备、器械和技术的进步，单孔与RATS的结合，被认为是未来的发展方向之一。

## 5 人体工程学

人体工程学是研究" 人-机-环境" 系统中人、机和环境三大要素之间的关系，为系统中的人的效能、健康问题提供理论与方法的学科，被应用到外科尤其是微创腔镜手术中。腔镜手术医生工作姿势不当会导致疲劳，进而可能会影响手术安全和医生健康。医生和医院管理者应该关注腔镜手术工程学问题，兼顾手术医生的操作舒适性，提高手术效率。有研究认为单孔与多孔VATS在外科人体工程学方面至少是等效的，但这方面的研究证据还有不足^[10, 11]^。而且既往相关研究多关注的是主刀医师，助手的舒适性则往往被忽略了。

## 6 美观

客观地讲，切口小而少，美观度自然会改善。但对于肿瘤患者特别是肺癌的治疗，美观的重要性一般会被放在相对次要的位置。单孔较多孔，美观的差异也相对很小。

## 7 总结

已有的研究显示单孔VATS肺切除手术是可行的，它对胸外科医生技术上提出了新的要求。现实世界中，单孔、两孔、三孔和四孔VATS并存，哪种径路肿瘤学效果更好？更安全？更微创？疼痛更轻？这还需要我们进一步研究，因为截至目前还没有确定的证据能用来进行最佳VATS肺切除径路的推荐。

手术技术的不断改进，精细手术器械的诞生及耗材成本的下降，将使胸腔镜手术不断进步。切口的大小只是一个方面，外科医生在依据自己对VATS技术熟悉度和掌握度的前提下，还要综合考量切口、器官和系统损伤。技术的可行性不等于治疗的合理性，在追求技术进步的同时，外科医生始终不能忘记安全、规范这一前提。
